# Ceramide Content in Liver Increases Along with Insulin Resistance in Obese Patients

**DOI:** 10.3390/jcm8122197

**Published:** 2019-12-12

**Authors:** Hady Razak Hady, Agnieszka U. Błachnio-Zabielska, Łukasz Szczerbiński, Piotr Zabielski, Monika Imierska, Jacek Dadan, Adam J. Krętowski

**Affiliations:** 1 1st Department of General Surgery and Endocrinology, Medical University Bialystok, 15-276 Bialystok, Poland; hadyrazakh@wp.pl (H.R.H.); jacdad@poczta.onet.pl (J.D.); 2 Department of Hygiene, Epidemiology and Metabolic Disorders, Medical University of Bialystok, 15-222 Bialystok, Poland; m.imierska@gmail.com; 3 Department of Endocrinology, Diabetology and Internal Medicine, Medical University of Bialystok, 15-276 Bialystok, Poland; lukasz.szczerbinski@umb.edu.pl (Ł.S.); adamkretowski@wp.pl (A.J.K.); 4 Department of Medical Biology, Medical University of Bialystok, 15-222 Bialystok, Poland; piotr.zabielski@umb.edu.pl; 5 Clinical Research Centre, Medical University of Bialystok, 15-276 Bialystok, Poland

**Keywords:** liver, obesity, bariatric surgery, ceramide, mass spectrometry: insulin resistance

## Abstract

The liver plays a central role in the glucose and lipid metabolism. Studies performed on animal models have shown an important role of lipid accumulation in the induction of insulin resistance. We sought to explain whether in obese humans, the insulin resistance is associated with hepatic ceramide accumulation. The experiments were conducted on obese men and women. Each gender was divided into three groups: Normal glucose tolerance group (NGT), Impaired glucose tolerance group (IGT), and Type 2 diabetic subjects (T2D). Ceramide (Cer) content was analyzed with the use of LC/MS/MS. An oral glucose tolerance test (OGTT), glycosylated hemoglobin (HbA1c), percentage body fat (FAT%), and body mass index (BMI) was also measured. Total hepatic ceramide was significantly higher in T2D females as compared to NGT females (*p <* 0.05), whereas in males, total ceramide was significantly higher in IGT and T2D as compared to NGT (*p <* 0.05). In both, men and women, the highest increase in T2D subjects, was observed in C16:0-Cer, C18:0:-Cer, C22:0-Cer, and C24:0-Cer (*p <* 0.05) as compared to NGT group. Interestingly, glucose (at 0′ and at 120′ in OGTT) and HbA1c positively correlated with the ceramide species that most increased in T2D patients (C16:0-Cer, C18:0-Cer, C22:0-Cer, and C24:0-Cer). In men glucose and HbA1c significantly correlated with only C22:0-Cer. This is one of the few studies comparing hepatic ceramide content in severely obese patients. We found that, ceramide content increased in diabetic patients, both in men and women, and the content of ceramide correlated with glycemic parameters. These data indicate ceramide contribution to the induction of hepatic insulin resistance.

## 1. Introduction

Obesity is defined as an excess of body fat. This state is associated with lipids accumulation not only in adipose tissue, but also in other tissues, such as skeletal muscle and the liver. Lipids storage in non-adipose tissue is thought to lead to several metabolic disturbances, including insulin resistance, type 2 diabetes (T2D) and cardiovascular disease [1,2,3,4]. Obesity has reached epidemic proportions worldwide, therefore insulin resistance and type 2 diabetes have become one of the most common chronic metabolic disorders. Therefore, it is important to get the knowledge about the mechanism of induction of insulin resistance. The liver is a key organ responsible for both glucose and lipid metabolism. Under physiological conditions, insulin inhibits hepatic gluconeogenesis, thereby preventing excessive postprandial glucose production. Hepatic insulin resistance is mainly manifested by ineffective inhibition of the gluconeogenesis by insulin, which in consequence leads to an increase in blood glucose level. Excessive intrahepatic lipids accumulation is closely related to the occurrence of insulin resistance and non-alcoholic fatty liver disease (NAFLD) [1,2,3]. Most publications regarding hepatic insulin resistance and NAFLD in association with hepatic lipids, refer to triacylglycerols and cholesterol, which accumulate in the liver in the largest amount [5,6]. Less information is available on liver bioactive lipids such as ceramide (Cer), which has been shown to play an important role in the induction of insulin resistance, first documented in skeletal muscle [7,8,9,10]. It has been repeatedly observed that Cer have the ability to inhibit the insulin pathway through activation of protein phosphatase-2A (PP2A), thus maintaining protein kinase B/Akt (PKB/Akt) in an unphosphorylated state [11,12,13]. The first paper presenting the relationship between ceramide accumulation and hepatic insulin resistance was published in 1990 [14]. Most studies on hepatic insulin resistance have been conducted in animal models. However, the data are rather ambiguous. In some works, both short- and long-term feeding with a high fat diet did not lead to an increase of ceramides in the liver, despite the induction of insulin resistance [15,16,17]. However, in most studies performed on animals, ceramide and other lipids content increases along with obesity, high fat diet feeding, or lard oil infusion [18,19,20,21,22], which is usually accompanied by a decrease in insulin sensitivity. However, there is still an unsolved mystery whether hepatic insulin resistance in humans is associated with the accumulation of ceramide in the liver. The few works performed on the human liver are inconclusive. In studies conducted on obese people without diabetes, it was found that the ceramide content did not significantly correlate with the Homeostatic Model Assessment index (HOMA-IR) [23]. On the other hand, recently published lipidomic data from obese humans showed a strong relationship between hepatic ceramide content and HOMA-IR value [24]. Therefore, the objective of the present study was to determine whether hepatic ceramide content is implicated in development of glucose metabolism disorders, including insulin resistance and T2D in obese men and woman with different levels of glucose metabolism disturbances. Our secondary goal was to observe any gender-specific differences between metabolic disturbances and hepatic ceramides.

## 2. Materials and Methods

### 2.1. Study Design and Population

The study included 130 obese patients (body mass index (BMI) > 40 kg/m^2^); 61 females (aged 47.6 ± 11.6) and 69 males (aged 44.1 ± 12.9) from Bialystok Bariatric Surgery Study (BBSS) [25] who underwent elective bariatric surgery in accordance with the National Institutes of Health guidelines for bariatric surgery [26]. Exclusion criteria were as follows: no prior bariatric surgery, gastrectomy, substance abuse, uncontrolled psychiatric illness, expected lack of compliance, or advanced stage cancer. All subjects gave their informed consent for inclusion before participating in the study. The study was conducted in accordance with the Declaration of Helsinki, and the protocol was approved by the Ethics Committee of the Medical University of Bialystok (Project identification code: R-I-002/546/2015). Female group included 30 patients with normal glucose tolerance (NGT group), 12 patients with impaired glucose tolerance (IGT group), and 19 patients with type 2 diabetes (T2D). Male group included 36 patients with NGT, 19 patients with IGT, and 14 patients with T2D (Table 1). Inclusion criteria for NGT, IGT, and T2D groups were applied according to American Diabetes Association guidelines: NGT group—HbA1c *<* 5.7, one-point oral glucose tolerance test (OGTT) at 120 min < 140 mg/dL; IGT group—5.7 < HbA1c < 6.5, 140 mg/dL < OGTT at 120 min < 200 mg/dL, no T2D diagnosis and no prior medication; T2D group HbA1C > 6.5, OGTT at 120 min > 200 mg/dL, prior T2D diagnosis and medication. Diabetic patients on oral antidiabetics were instructed not to take medication 24 h before the day of surgery and glucose concentration was monitored and regulated individually by intravenous insulin infusion or repeated doses of rapid-acting insulin, as requested by anesthesiologist. Diabetic patients on insulin treatment were continuing their regimen, with the individualized adjustment of the dose. The liver samples were collected during bariatric surgery procedure, performed at the First Clinical Department of General and Endocrine Surgery at the Medical University of Bialystok. The liver samples were immediately snap-frozen after collection and then stored in liquid nitrogen for further analysis.

The study included 130 obese patients (body mass index (BMI) > 40 kg/m^2^); 61 females (aged 47.6 ± 11.6) and 69 males (aged 44.1 ± 12.9) from Bialystok Bariatric Surgery Study (BBSS) [25] who underwent elective bariatric surgery in accordance with the National Institutes of Health guidelines for bariatric surgery [26]. Exclusion criteria were as follows: no prior bariatric surgery, gastrectomy, substance abuse, uncontrolled psychiatric illness, expected lack of compliance, or advanced stage cancer. All subjects gave their informed consent for inclusion before participating in the study. The study was conducted in accordance with the Declaration of Helsinki, and the protocol was approved by the Ethics Committee of the Medical University of Bialystok (Project identification code: R-I-002/546/2015). Female group included 30 patients with normal glucose tolerance (NGT group), 12 patients with impaired glucose tolerance (IGT group), and 19 patients with type 2 diabetes (T2D). Male group included 36 patients with NGT, 19 patients with IGT, and 14 patients with T2D (Table 1). Inclusion criteria for NGT, IGT, and T2D groups were applied according to American Diabetes Association guidelines: NGT group—HbA1c *<* 5.7, one-point oral glucose tolerance test (OGTT) at 120 min < 140 mg/dL; IGT group—5.7 < HbA1c < 6.5, 140 mg/dL < OGTT at 120 min < 200 mg/dL, no T2D diagnosis and no prior medication; T2D group HbA1C > 6.5, OGTT at 120 min > 200 mg/dL, prior T2D diagnosis and medication. Diabetic patients on oral antidiabetics were instructed not to take medication 24 h before the day of surgery and glucose concentration was monitored and regulated individually by intravenous insulin infusion or repeated doses of rapid-acting insulin, as requested by anesthesiologist. Diabetic patients on insulin treatment were continuing their regimen, with the individualized adjustment of the dose. The liver samples were collected during bariatric surgery procedure, performed at the First Clinical Department of General and Endocrine Surgery at the Medical University of Bialystok. The liver samples were immediately snap-frozen after collection and then stored in liquid nitrogen for further analysis.

### 2.2. Body Composition Analysis

Whole body dual energy X-ray absorptiometry (DXA) scans were performed for analysis of body composition, using Lunar iDXA (GE Healthcare, Chicago, IL, USA).

### 2.3. Oral Glucose Tolerance Test (OGTT)

OGTT was conducted according to the recommendations of the American Diabetes Association (ADA). OGTT test was performed during recruitment phase for BBSS program to gather additional data on severity of metabolic dysregulation and were not performed for diagnostic purposes. OGTT was performed under the guidance of a diabetologist, under informed consent and in accordance with R-I-002/546/2015 Ethics Committee approval. Patients were instructed not to take any medications on the day of the test. The test was performer in the morning after an overnight fast of 8–10 h. Patients were instructed to avoid intense exercise for 24 h before the test. A blood sample was collected at baseline and then the patient consumed the 75 g glucose in 300 mL of lukewarm water. Further blood samples were collected 30, 60, and 120 min after the glucose administration.

### 2.4. Sample Preparation and Laboratory Measurements

All samples were collected from patients in the overnight fasting state. Blood samples were collected before surgery to ethylenediaminetetraacetic acid (EDTA)-coated tubes and centrifuged for 10 min at 4000 rpm. glucose and insulin were quantified by using an Abbott analyzer (Abbott Diagnostics, Wiesbaden, Germany). At the end of the surgical intervention, the samples of liver were taken and promptly frozen in liquid nitrogen and stored at −80 °C until future analyzes.

### 2.5. Sphingolipid Measurements

The sphingolipids content was measured using a ultra-high performance liquid chromatography-tandem mass spectrometry (UHPLC/MS/MS) approach according to Blachnio-Zabielska et al. [27]. Briefly, liver samples (~20 mg) were pulverized in LN_2_ homogenized in a solution composed of 0.25 M sucrose, 25 mM KCl, 50 mM Tris, and 0.5 mM EDTA, pH 7.4. Afterwards, the internal standard solution (C15-d7-Cer, C16:0-d7-Cer, C18:0-d7-Cer, C24:0-d7-Cer, C24:1-d7-Cer, d17:1/18:1-Cer, d17:1/20:0-Cer, Avanti Polar Lipids, Alabaster, AL, USA) as well as extraction mixture (isopropanol:water:ethyl acetate, 30:10:60; *v*/*v*/*v*) were added to each sample. The mixture was vortexed, sonicated and centrifuged for 10 min at 4000 g (MPW 350R). The supernatant was transferred to a new vial and pellet was re-extracted with the same extraction mixture. After centrifugation, supernatants were combined and evaporated under nitrogen. Then, samples were reconstituted in LC Solvent B (2 mM ammonium formate, 0.1% formic acid in methanol. Ceramides were analyzed with the use of a Sciex QTRAP 6500 + triple quadrupole mass spectrometer, using positive ion electrospray ionization (ESI) source (except of S1P, which was analyzed in negative mode) with multiple reaction monitoring (MRM) against standard curves constructed for each compound. The chromatographic separation was performed using reverse-phase column (Zorbax SB-C8 column 2.1 × 150 mm, 1.8 μm) (Agilent, Santa Clara, CA, USA). Chromatographic separation was conducted in binary gradient using 1 mM ammonium formate, 0.1% formic acid in water as solvent A and 2 mM Ammonium formate, 0.1% formic acid in methanol as solvent B at the flow rate of 0.4 mL/min.

### 2.6. Statistical Significance Estimation and Correlation Analysis

Statistical significance between experimental groups was estimated using ANOVA with Tukey Honest Significant Difference HSD post-hoc test for unequal n-numbers. Significance level was set to *p* < 0.05. We used Pearson’s r approach with Bonferroni correction for multiple comparisons (corrected *p*-value < 0.00095) to establish relationships between selected variables.

## 3. Results

### 3.1. Participant Demographic and Anthropometric Information

Within females and males groups, there were no significant differences in percentage body fat (%FAT) between NGT vs. IGT vs. T2D (Table 1). Fasting glucose concentration was significantly higher in T2D group in both females and males, as compared to NGT and IGT groups (*p* < 0.05). Although the increase in fasting plasma insulin in both IGT and T2D groups (males and females) was not significant, the HOMA-IR coefficient was significantly higher in T2D subjects in both, male and female groups, as compared to respective NGT group (*p* < 0.05). Moreover, HOMA-IR in IGT females was significantly higher than in NGT (*p* < 0.05). In 120 min after glucose ingestion, the blood glucose concentration was significantly higher in IGT as compared to NGT (in both females and males *p* < 0.005) as well as in T2D groups in females and males as compared to IGT and NGT (*p* < 0.05). The glycosylated hemoglobin HbA1c in females was significantly higher in T2D subjects as compared to NGT and IGT groups (*p* < 0.05) whereas in males HbA1c was significantly higher in IGT comparing to NGT (*p* < 0.05) as well as in T2D as compared to NGT and IGT (*p* < 0.05).

### 3.2. Hepatic Ceramide Content

**Figure 1:**
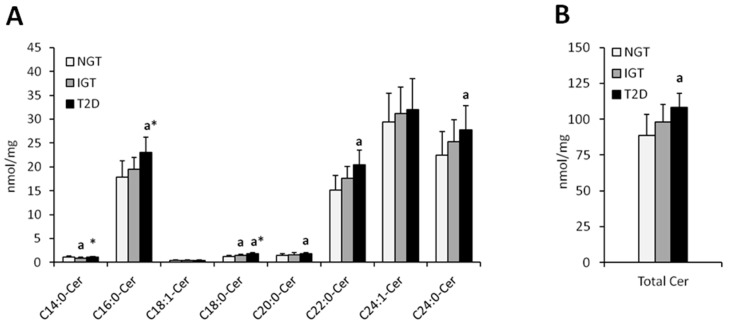
Concentration of individual (Panel (**A**)) and total (Panel (**B**)) hepatic ceramides in obese females. NGT—normal glucose tolerance group, IGT—impaired glucose tolerance group; T2D—type 2 diabetes group. Values are mean ± standard deviation; ^a^ *p* < 0.05 vs. NGT, * *p* < 0.05.

**Figure 2:**
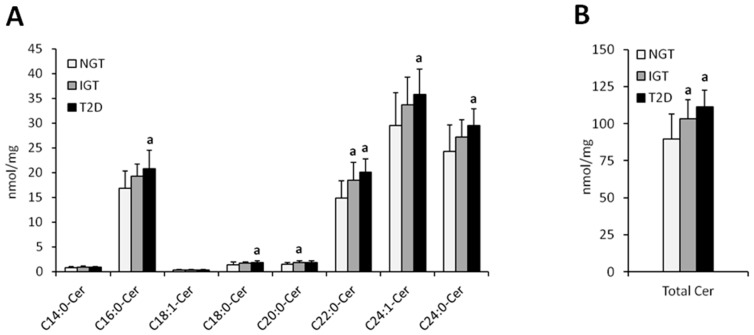
Concentration of individual (Panel (**A**)) and total (Panel (**B**)) hepatic ceramides in obese males. NGT—normal glucose tolerance group, IGT—impaired glucose tolerance group; T2D—type 2 diabetes group. Values are mean ± standard deviation; ^a^ *p* < 0.05 vs. NGT, * *p* < 0.05.

Total hepatic ceramide content was significantly higher in T2D females as compared to NGT females (*p* < 0.05) whereas in males, total ceramide content was significantly higher in IGT and T2D as compared to NGT (*p* < 0.05) (Figure 1 and Figure 2, Table 2). In both, men and women, the highest increase in T2D subjects as compared to NGT group, was observed in C16:0-Cer, C18:0:-Cer, C22:0-Cer, and C24:0-Cer (*p* < 0.05). In woman, significant elevation in T2D group was also noticed in C14:0-Cer and C20:0-Cer (*p* < 0.05), in comparison to NGT group. In men, significant increase in T2D patients was observed in C24:1-Cer (*p* < 0.05) as compared to NGT. Moreover, in males, in IGT group, there was significant increase in C20:0-Cer, C22:0-Cer, and total ceramide as compared to NGT subjects (*p* < 0.05). Analysis performed on combined data (irrespective of gender) yielded similar results (Figure S1), with the increase in C22:0-Cer and total Cer in combined T2D group reaching significance compared to both the NGT and IGT group as the only difference not observed in separate analysis.

### 3.3. Correlations between Anthropometric Parameters and Hepatic Ceramides

**Figure 3:**
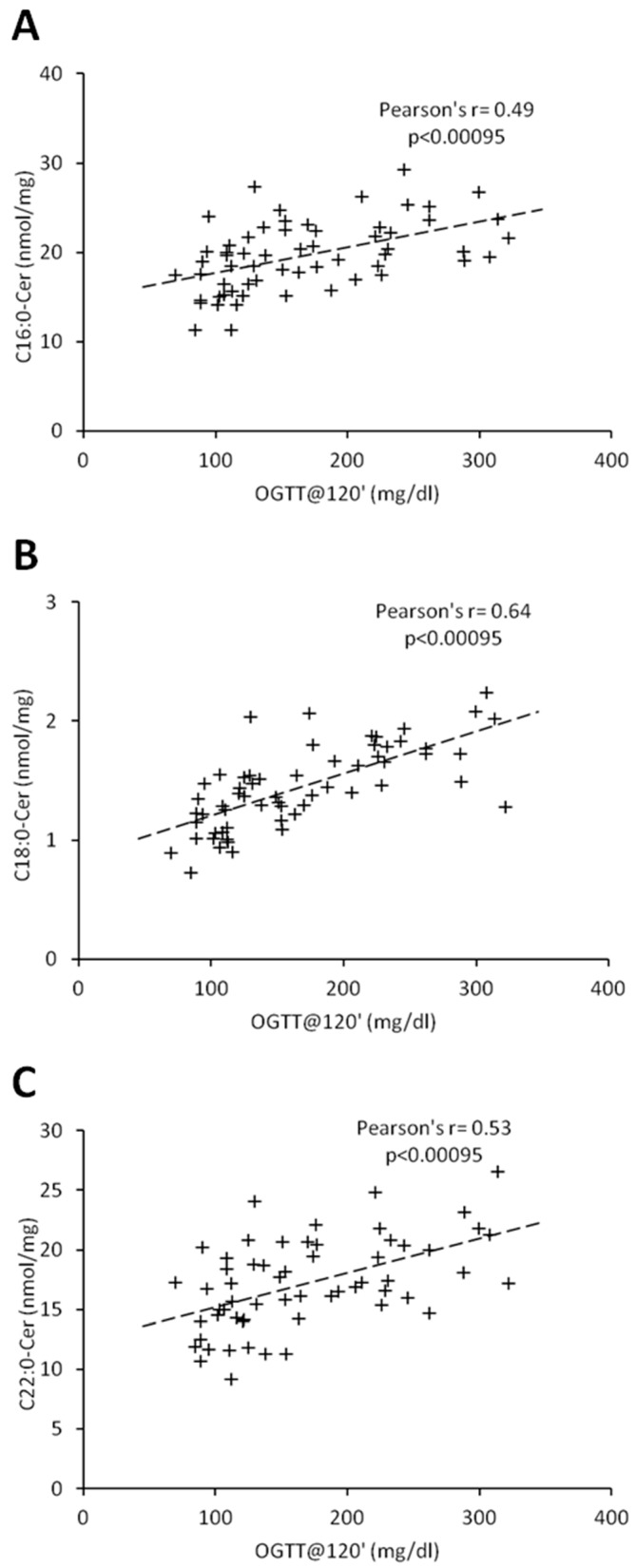
Correlation of C16:0 (Panel (**A**)), C18:0-Cer (Panel (**B**)), and C22:0-Cer (Panel (**C**)) with blood plasma glucose concentration at 120 min of OGTT test in females. Pearson’s r correlation coefficient and correlation significance is given in graph inserts. OGTT—oral glucose tolerance test.

**Figure 4:**
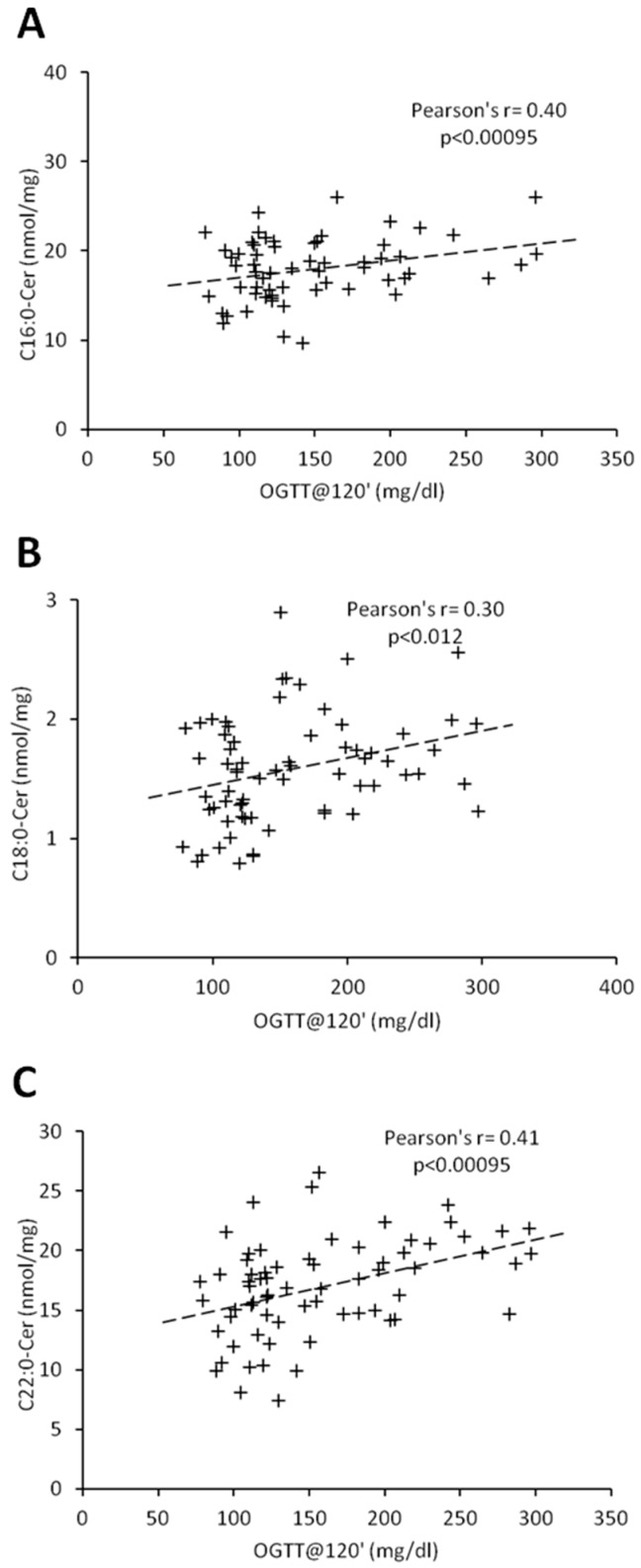
Correlation of C16:0 (Panel (**A**)), C18:0-Cer (Panel (**B**)), and C22:0-Cer (Panel (**C**)) with blood plasma glucose concentration at 120 min of OGTT test in males. Pearson’s r correlation coefficient and correlation significance is given in graph inserts. OGTT—oral glucose tolerance test.

Percentage of body fat and C18:1-Cer positively correlated in both females and males (r = 0.45, *p* < 0.00095; r = 0.54, *p* < 0.00095, respectively) (Table 3 and Table 4). In women, there was no significant correlation between ceramides and BMI, whereas in men, we observed a positive correlation between BMI and C18:1-Cer (r = 0.45, *p* < 0.00095). Interestingly, in females, glycemic parameters such as fasting glucose concentration, glucose concentration 120 min after glucose administration (OGTT at 120′) and HbA1c correlated significantly with the ceramides which were significantly elevated in the T2D group (C16:0-Cer, C18:0-Cer and C22:0-Cer) (Table 2 and Table 3). Both the C20:0-Cer and total hepatic ceramide displayed positive correlation with OGTT at 120′ value. In man, all three glycemic parameters significantly correlated only with C22:0-Cer (Table 4). Moreover, glucose concentration 120 min after glucose administration significantly correlated with both the C16:0-Cer and total Cer. None of the measured ceramide species displayed significant correlation with HOMA-IR value in both females and males. Among all the measured glycemic parameters, plasma glucose concentration at 120 min of OGTT test (OGTT at 120′) showed highest number of significant correlations with hepatic ceramide species. Figure 3 and Figure 4 show individual correlations between plasma glucose at OGTT at 120′ and C16:0-Cer, C18:0-Cer and C22:0-Cer in both females and males, respectively. Combining both groups led to decrease of individual ceramide Pearson’s r correlation strength, as compared to female-only correlations, but to increase of correlation strength as compared to male-only data with FAT%(DXA) and C18:1-Cer being a sole exception (Table 3 and Table 4, Table S1, Figure S2). It has to be noted though that correlations of C20:0-Cer and total ceramide concentration and OGTT at 0’, OGTT at 120′, and HbA1c reached significance level despite decrease in the correlation strength as compared with female-only data.

## 4. Discussion

Obesity is associated with an increased risk of non-alcoholic fatty liver disease, characterized by an elevated intrahepatic lipid content and severe metabolic disorders, including insulin resistance, type 2 diabetes, and metabolic syndrome. The mechanism of defects in hepatic insulin-signaling cascade has not been thoroughly understood, but it has been well documented that hepatic insulin resistance is associated with an increased hepatic lipid accumulation [28,29,30]. For many years, the attention of scientists was focused on the participation of triacylglycerols in hepatic insulin resistance, but studies performed on animal have demonstrated that biologically active lipids such as ceramides, can be directly involved in induction of liver metabolic abnormalities [18,19,20,21,22]. However, there are conflicting data regarding the role of ceramide in the induction of hepatic insulin resistance. In genetically obese Zucker diabetic fatty (ZDF) rats [22] or in mice fed high-fat diet (HFD) [17] no increase in total hepatic ceramide was observed despite the induction of glucose metabolism disorders. However, most studies have shown that HFD leads to an increase in liver ceramide levels, accompanied by a decrease in insulin sensitivity [18,19,20,21,22]. In study performed on C57BL/6N mice fed HFD diet for 14 weeks, a significant increase in C14:0-Cer, C16:0-Cer, C18:0-Cer, C20:0-Cer, and C24:1-Cer was observed [21]. In another study, in which C57BL/6 mice were fed HFD for 16–17 weeks, a significant elevation of C16:0-Cer, C20:0-Cer, and C22:0-Cer was noticed, and these changes were accompanied by impaired glucose tolerance [31]. Moreover, in leptin-deficient (*ob*/*ob*) mice, (a model of type 2 diabetes), an increased total hepatic ceramide content has been also found [32]. We have previously demonstrated that in Wistar rats fed HFD for 8 weeks, the increase in hepatic content of individual ceramide species (C16:0-Cer, C18:0-Cer, C20-Cer, C22:0-Cer, and C24:0-Cer) was associated with elevated HOMA-IR value [18,19]. Interestingly, the adverse effects were abolished after using myriocin, an inhibitor of sphingolipid de novo synthesis [19,20,22,33]. Studies using genetically modified mice, lacking the gene encoding ceramide synthase 6 (CerS6), that specifically synthesizes palmitoyl-ceramide, have shown that C16:0-Cer plays a particularly important role in hepatic insulin resistance [34]. It has been found that the CerS6 knockout mice were protected from the development of obesity when fed a high-fat diet [21,34]. In addition, overexpression of liver-specific ceramidase, the enzyme responsible for ceramide hydrolysis, reduced liver ceramide content, which protected mice fed a high-fat diet from fatty liver and was associated with improved insulin sensitivity [35]. Above animal studies underline the importance of ceramide accumulation in the induction of hepatic insulin resistance. Moreover, decrease in the liver ceramide by chemical CerS inhibitors [19], gene knock-out [21,34], or dietary intervention in HFD-fed obese animals [36] was accompanied by restoration of whole body or hepatic insulin sensitivity. The data presented suggest that the specific C16: 0-Cer ceramide produced by CerS6 plays an important role in the development of hepatic insulin resistance, therefore it can be assumed that the inhibition of CerS6-mediated ceramide C16: 0-Cer synthesis may be a potentially attractive target in the treatment of insulin resistance in obesity and T2D. Although most of these studies have been performed in animal models, recent human lipidomic data have shown a strong relationship between hepatic ceramides and the HOMA-IR index [24]. However, most of the work investigating the role of lipids in induction of insulin resistance compare obese or HFD fed subjects with lean counterparts and it is not surprising, that with the weight gain, the amount of lipids increases in various tissues, including the liver [7,10,18,19,21,22,37,38,39]. Our work is one of the first that compares hepatic ceramide content in severely obese mean and women with different degree of glucose metabolism disorder. In our view the most interesting finding of the study is that individual hepatic long-chain ceramides increase with the severity of glucose metabolism abnormalities, in both men and women, despite the same average percentage of body fat and BMI. These findings highlight the importance of hepatic ceramide accumulation in the induction of insulin resistance. Moreover, despite universal nature of this observation across men and women, females displayed greater correlation between individual hepatic ceramides and indices of insulin resistance, as shown in the case of C18:0-Cer. This particular ceramide species was previously identified as a key player in the induction of skeletal muscle insulin resistance [7,40]. This points toward possible gender differences in hepatic ceramide accumulation. To our knowledge, only two other studies related to human insulin resistance and obesity were targeting hepatic ceramides. The study by Sajan et al. [41] was performed on cryopreserved liver samples from liver transplant donors with varying degree of obesity and diabetic status. Both short-chain and long-chain ceramide species accumulated in livers of obese and T2D subjects with C14:0-Cer and C16:0-Cer positively correlating with BMI values. Moreover, the authors showed that increased ceramide concentration was accompanied by activation of atypical PKC isoform (aPKC, molecular target of ceramide) and with decreased phosphorylation of Akt. Due to the fact that all the study subjects from Sajan et al. work were of mixed gender and ethnicity and were on life support with parenteral hypocaloric nutrition, the results cannot be directly compared with our work. A more recent study by Apostolopoulou et al., performed on obese subjects with various stages of NAFLD or steatohepatitis, did not show considerable alternations in individual liver ceramides [42]. Compared to our study, subjects were of similar age and BMI, yet did not display similar alterations in individual ceramide species, except C24:0-Cer. Most notable changes in insulin-resistant NASH group were noted for ceramide species which does not possess biological activity (sphinganine-based C16:0; C22:0, and C24:0 dihydroceramides) or the products of complex sphingolipid degradation (long-chain lactosyl- and hexosyl-ceramides). The only similarity with our findings is the gradual elevation of hepatic total ceramide parallel to the increasing hepatic steatosis, yet no significant correlations with indices of insulin resistance were found. However, the number of subjects in each group was small (*n* = 7), which could have caused a lack of statistically significant differences. In the present study, we have found that total ceramide elevation was accompanied by an increase in the HOMA-IR index. We have observed significant increase in C16:0-Cer, C18:0-Cer, C22:0-Cer, and C24:0-Cer, in both, men and women in subjects with diagnosed T2D, despite similar adiposity and BMI as compared to both NGT and IGT groups. Moreover, we had shown that the hepatic concentration of individual ceramide species correlates significantly with glycemic parameters i.e., fasting glucose, OGTT results, and HbA1C value. Moreover, our data point at possible gender-specific differences in the relationship between ceramide and insulin-resistance, as we observed higher number of altered ceramide species and respective correlations in women than in men. We also observed an increased content of individual ceramides in the IGT group. In women, it was only C18:0-Cer but in men it was C20:0-Cer and C22:0-Cer. These results show that the individual ceramide content in the liver varies depending on the gender and severity of glucose metabolism disorders. 

## 5. Conclusions

Taking together, our work is one of the first to compare ceramide content in liver of severely obese men and women scheduled for bariatric surgery, with varying degree of glucose metabolism disturbances. We found that ceramide level increases with the severity of glucose metabolism dysregulation, both in men and women despite no difference in fat percentage and BMI. This underlines the importance of ceramide accumulation in hepatic insulin resistance. Moreover, we have demonstrated, that in females, glycemic parameters such as fasting glucose concentration, glucose concentration at 120 min in OGTT, and HbAc1, significantly correlated with those ceramide, which elevation was the highest in T2D patients, whereas in males, only C22:0-Cer significantly correlated with all the glycemic parameters. The data suggest, that ceramides may play significant role in progression of insulin resistance and that the impact of ceramide may differ between men and women.

## Figures and Tables

**Table 1:** Anthropometric parameters.

	Females			Males		
	NGT	IGT	T2D	NGT	IGT	T2D
Age (years)	42.6 ± 11.5	53.2 ± 8.3 ^a^	51.9 ± 10.5 ^a^	41.5 ± 13.0	43.0 ± 11.4	52.1 ± 12.4
BMI (kg/m^2^)	48.8 ± 7.99	44.1 ± 7.3	45.9 ± 7.33	46.6 ± 6.1	51.6 ± 8.6	49.7 ± 8.2
%FAT (DXA)	53.7 ± 4.03	50.2 ± 3.9	51.2 ± 3.6	46.1 ± 5.5	48.2 ± 4.5	45.7 ± 4.7
OGTT at 0′ (mg/dL)	107.3 ± 7.7	124.1 ± 13.3	164.7 ± 56.4 ^a,^*	110.0 ± 10.0	128.0 ± 19.2	170.1 ± 46.7 ^a,^*
OGTT at 120′ (mg/dL)	113.9 ± 22.6	189.5 ± 34.6 ^a^	258.1 ± 85.5 ^a,^*	115.4 ± 23.2	178.7 ± 39.1 ^a^	240.1 ± 41.2 ^a,^*
HbA1c (%Hb)	5.58 ± 0.32	6.2 ± 0.62	7.2 ± 1.4 ^a,^*	5.6 ± 0.4	6.2 ± 0.5 ^a^	7.3 ± 1.1 ^a,^*
HOMA-IR	4.90 ± 2.62	8.52 ± 2.56 ^a^	9.96 ± 4.26 ^a^	6.50 ± 2.82	12.1 ± 11.20	13.30 ± 9.23 ^a^
Insulin (mU/mL)	0.018 ± 0.009	0.027 ± 0.006	0.025 ± 0.014	0.024 ± 0.010	0.036 ± 0.030	0.030 ± 0.015

Data expressed as mean ± standard deviation; NGT—normal glucose tolerance group; IGT—impaired glucose tolerance group; T2D—type 2 diabetes group; BMI—body mass index; FAT% (DXA)—percentage of body fat as measured by dual-energy X-ray absorptiometry; OGTT—oral glucose tolerance test (values for 0 min and 120 min); HbA1c—percentage of glycated hemoglobin; HOMA-IR—homeostatic model assessment of insulin resistance. ^a^ *p* < 0.05 vs. NGT; * *p* < 0.05 vs. IGT.

**Table 2:** Concentration of individual ceramide molecular species in liver of obese females and males.

Ceramide nmol/mg	Females			Males		
	NGT	IGT	T2D	NGT	IGT	T2D
**C14:0-Cer**	1.08 ± 0.23	0.89 ± 0.16 ^a^	1.15 ± 0.12 *	0.81 ± 0.23	0.94 ± 0.19	0.92 ± 0.12
**C16:0-Cer**	17.81 ± 3.4	19.49 ± 2.5	22.99 ± 3.1 ^a,^*	16.83 ± 3.50	19.25 ± 2.45	20.84 ± 3.71 ^a^
**C18:1-Cer**	0.43 ± 0.09	0.40 ± 0.11	0.38 ± 0.08	0.33 ± 0.11	0.36 ± 0.10	0.35 ± 0.09
**C18:0-Cer**	1.21 ± 0.22	1.52 ± 0.17 ^a^	1.80 ± 0.25 ^a,^*	1.42 ± 0.48	1.68 ± 0.32	1.86 ± 0.36 ^a^
**C20:0-Cer**	1.46 ± 0.30	1.62 ± 0.43	1.80 ± 0.28 ^a^	1.48 ± 0.36	1.83 ± 0.33 ^a^	1.79 ± 0.35
**C22:0-Cer**	15.11 ± 3.13	17.59 ± 2.47	20.48 ± 3.04 ^a^	14.87 ± 3.54	18.49 ± 3.59 ^a^	20.16 ± 2.67 ^a^
**C24:1-Cer**	29.37 ± 6.01	31.20 ± 5.54	32.03 ± 6.49	29.48 ± 6.73	33.68 ± 5.64	35.85 ± 5.09 ^a^
**C24:0-Cer**	22.40 ± 5.02	25.32 ± 4.55	27.74 ± 5.11 ^a^	24.25 ± 5.39	27.16 ± 3.49	29.59 ± 3.37 ^a^
**Total Cer**	88.87 ± 14.46	98.02 ± 12.22	108.37 ± 9.73 ^a^	89.47 ± 17.17	103.41 ± 12.54 ^a^	111.36 ± 11.15 ^a^

Data expressed as mean ± standard deviation; NGT—normal glucose tolerance group; IGT—impaired glucose tolerance group; T2D—type 2 diabetes group; ^a^ *p* < 0.05 vs. NGT; * *p* < 0.05 vs. IGT.

**Table 3:** Correlations between individual hepatic ceramide molecular species and selected anthropometric measurements in obese females.

	C14:0-Cer	C16:0-Cer	C18:1-Cer	C18:0-Cer	C20:0-Cer	C22:0-Cer	C24:1-Cer	C24:0-Cer	Total Cer
**OGTT at 0’**	*r* = 0.0275	*r* = 0.4278 ^a^	*r* = −0.2233	*r* = 0.4842 ^a^	0.3393	*r* = 0.442 ^a^	*r* = 0.1246	*r* = 0.1951	*r* = 0.3574
	*p* = 0.833	*p* = 0.000	*p* = 0.084	p = 0.000	*p* = 0.007	*p* = 0.000	*p* = 0.339	*p* = 0.132	*p* = 0.005
**OGTT at 120’**	*r* = 0.0226	*r* = 0.4915 ^a^	*r* = −0.1722	*r* = 0.6453 ^a^	*r* = 0.487 ^a^	*r* = 0.5263 ^a^	*r* = 0.1821	r = 0.2292	*r* = 0.4373 ^a^
	*p* = 0.863	*p* = 0.000	*p* = 0.185	p = 0.000	*p* = 0.000	*p* = 0.000	*p* = 0.160	*p* = 0.076	*p* = 0.000
**HbA1c**	*r* = 0.067	*r* = 0.4235 ^a^	*r* = −0.1776	*r* = 0.5082 ^a^	0.3688	*r* = 0.429 ^a^	*r* = 0.139	*r* = 0.1661	*r* = 0.3505
	*p* = 0.608	*p* = 0.000	*p* = 0.171	*p* = 0.000	*p* = 0.003	*p* = 0.000	*p* = 0.285	*p* = 0.201	*p* = 0.006
**BMI**	*r* = 0.2654	*r* = 0.01	*r* = 0.2773	*r* = −0.0148	*r* = 0.0187	*r* = −0.0513	*r* = 0.0265	*r* = 0.0897	*r* = 0.0381
	*p* = 0.039	*p* = 0.939	*p* = 0.030	*p* = 0.910	*p* = 0.886	*p* = 0.695	*p* = 0.840	*p* = 0.492	*p* = 0.771
**FAT% (DXA)**	*r* = 0.2661	*r* = −0.0967	*r* = 0.4508 ^a^	*r* = −0.1051	*r* = 0.0605	*r* = −0.1082	*r* = 0.0542	*r* = 0.0816	*r* = 0.0048
	*p* = 0.038	*p* = 0.458	*p* = 0.000	*p* = 0.420	*p* = 0.643	*p* = 0.407	*p* = 0.678	*p* = 0.532	*p* = 0.971
**HOMA-IR**	*r* = −0.1504	*r* = 0.1348	*r* = −0.2186	*r* = 0.3687	*r* = 0.2816	*r* = 0.2056	*r* = 0.1406	*r* = 0.0445	*r* = 0.1684
	*p* = 0.318	*p* = 0.372	*p* = 0.144	*p* = 0.012	*p* = 0.058	*p* = 0.171	*p* = 0.351	*p* = 0.769	*p* = 0.263

Values show Pearson’s r correlation coefficient together with correlation *p*-value. Correlations in bold type are significant with *p* < 0.00095 (*p*-value of 0.05 after Bonferroni correction for multiple comparisons); OGTT—oral glucose tolerance test (values for 0 min and 120 min); HbA1c—glycated hemoglobin; BMI—body mass index; FAT% (DXA)—percentage of body fat as measured by dual-energy X-ray absorptiometry; HOMA-IR—homeostatic model assessment of insulin resistance; ^a^ *p* < 0.00095.

**Table 4:** Correlations between individual hepatic ceramide molecular species and selected anthropometric measurements in obese males.

	C14:0-Cer	C16:0-Cer	C18:1-Cer	C18:0-Cer	C20:0-Cer	C22:0-Cer	C24:1-Cer	C24:0-Cer	Total Cer
**OGTT at 0’**	*r* = 0.0574	*r* = 0.3426	*r* = -0.0391	*r* = 0.1592	*r* = 0.2715	*r* = 0.4197 ^a^	*r* = 0.2046	*r* = 0.3566	*r* = 0.3613
	*p* = 0.640	*p* = 0.004	*p* = 0.750	*p* = 0.191	*p* = 0.024	*p* = 0.000	*p* = 0.092	*p* = 0.003	*p* = 0.002
**OGTT at 120’**	*r* = 0.0951	*r* = 0.3978 ^a^	*r* = 0.1038	*r* = 0.2999	*r* = 0.3384	*r* = 0.4114 ^a^	*r* = 0.2681	*r* = 0.3486	*r* = 0.3994 ^a^
	*p* = 0.437	*p* = 0.000	*p* = 0.396	*p* = 0.012	*p* = 0.004	*p* = 0.000	*p* = 0.026	*p* = 0.003	*p* = 0.000
**HbA1c**	*r* = 0.1008	*r* = 0.356	*r* = -0.0491	*r* = 0.3009	*r* = 0.3009	*r* = 0.3953 ^a^	*r* = 0.1997	*r* = 0.3749	*r* = 0.3666
	*p* = 0.410	*p* = 0.003	*p* = 0.689	*p* = 0.012	*p* = 0.012	*p* = 0.000	*p* = 0.100	*p* = 0.002	*p* = 0.002
**BMI**	*r* = 0.2188	*r* = 0.3048	*r* = 0.4503 ^a^	*r* = 0.3253	*r* = 0.3184	*r* = 0.2292	*r* = 0.2589	*r* = 0.0318	*r* = 0.2463
	*p* = 0.071	*p* = 0.011	*p* = 0.000	*p* = 0.006	*p* = 0.008	*p* = 0.058	*p* = 0.032	*p* = 0.795	*p* = 0.041
**FAT% (DXA)**	*r* = 0.2853	*r* = 0.2735	*r* = 0.5358 ^a^	*r* = 0.247	*r* = 0.2622	*r* = 0.1707	*r* = 0.2577	*r* = 0.1113	*r* = 0.2466
	*p* = 0.017	*p* = 0.023	*p* = 0.000	*p* = 0.041	*p* = 0.029	*p* = 0.161	*p* = 0.033	*p* = 0.363	*p* = 0.041
**HOMA-IR**	*r* = 0.062	*r* = 0.2999	*r* = 0.1268	*r* = 0.1512	*r* = 0.2754	*r* = 0.1757	*r* = 0.0719	*r* = 0.1531	*r* = 0.1797
	*p* = 0.669	*p* = 0.034	*p* = 0.380	*p* = 0.295	*p* = 0.053	*p* = 0.222	*p* = 0.620	*p* = 0.289	*p* = 0.212

Values show Pearson’s r correlation coefficient together with correlation *p*-value. Correlations in bold type are significant with *p* < 0.00095 (*p*-value of 0.05 after Bonferroni correction for multiple comparisons); OGTT—oral glucose tolerance test (0 min and 120 min); HbA1c—glycated hemoglobin; BMI—body mass index; FAT% (DXA)—percentage of body fat as measured by dual-energy X-ray absorptiometry; HOMA-IR—Homeostatic model assessment of insulin resistance; ^a^ *p* < 0.00095.

## Supplementary Materials

The following are available online at https://www.mdpi.com/2077-0383/8/12/2197/s1, Figure S1: Concentration of individual (Panel A) and total (Panel B) hepatic ceramides in combined group., Figure S2: Correlation of C16:0 (Panel A), C18:0-Cer (Panel B) and C22:0-Cer (Panel C) with blood plasma glucose concentration at 120 min of OGTT test in combined group., Table S1: Correlations between individual hepatic ceramide molecular species and selected anthropometric measurements in combined (both males and females) group.
