# Supplementary material for: Ceramide Content in Liver Increases Along with Insulin Resistance in Obese Patients
Source: J Clin Med. 2019 Dec 12;8(12):2197. doi: 10.3390/jcm8122197 (PMC6947381; doi:10.3390/jcm8122197)
Supplement: Supplementary file 1 [file jcm-08-02197-s001.pdf]

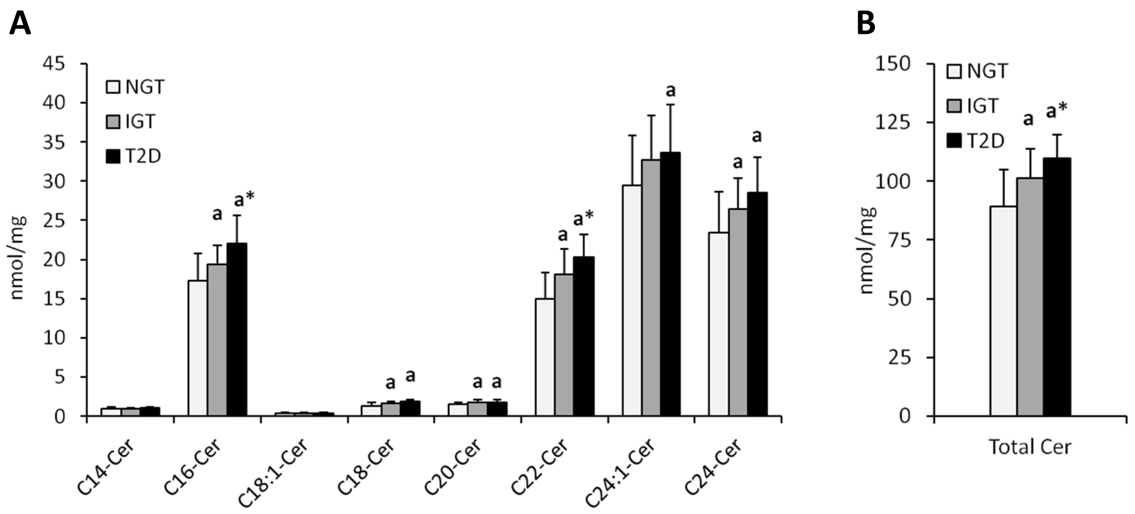

Figure S1. Concentration of individual (Panel A) and total (Panel B) hepatic ceramides in combined group. NGT – normal glucose tolerance group, IGT – impaired glucose tolerance group; T2D – type 2 diabetes group. Values are mean ± standard deviation; a – $p<0.05$ vs. NGT, * - $p<0.05$.

Table S1. Correlations between individual hepatic ceramide molecular species and selected anthropometric measurements in combined (both males and females) group.

	C14:0-Cer	C16:0-Cer	C18:1-Cer	C18:0-Cer	C20-Cer	C22:0-Cer	C24:1-Cer	C24:0-Cer	Total Cer
**OGTT at 0'**	r=0.0451	**r=0.3839**[a]	r=0.-1162	**r=0.2912**[a]	**r=0.3006**	**r=0.4272**[a]	r=0.1601	r=0.2634	**r=0.3536**[a]
	p=0.610	**p=0.000**	p=0.188	**p=0.001**	**p=0.001**	**p=0.000**	p=0.069	p=0.002	**p=0.000**
**OGTT at 120'**	r=0.0956	**r=0.4575**[a]	r=0.-0034	**r=0.4207**[a]	**r=0.3987**	**r=0.4649**[a]	r=0.2033	r=0.2580	**r=0.4020**[a]
	p=0.279	**p=0.000**	p=0.969	**p=0.000**	**p=0.000**	**p=0.000**	p=0.020	p=0.003	**p=0.000**
**HbA1c**	r=0.0929	**r=0.3922**[a]	r=0.-0924	**r=0.3718**[a]	**r=0.3277**	**r=0.4091**[a]	r=0.1614	r=0.2514	**r=0.3513**[a]
	p=0.293	**p=0.000**	p=0.296	**p=0.000**	**p=0.000**	**p=0.000**	p=0.067	p=0.004	**p=0.000**
**BMI**	r=0.1705	r=0.1350	**r=0.3184**[a]	r=0.1979	r=0.1867	r=0.0951	r=0.1626	r=0.0750	r=0.1559
	p=0.052	p=0.126	**p=0.000**	p=0.024	p=0.033	p=0.282	p=0.065	p=0.396	p=0.077
**FAT% (DXA)**	**r=0.4354**[a]	r=0.1924	**r=0.5685**[a]	r=0.0205	r=0.1236	r=0.0725	r=0.0940	r=0.0077	r=0.1150
	**p=0.000**	p=0.028	**p=0.000**	p=0.817	p=0.161	p=0.412	p=0.288	p=0.930	p=0.193
**HOMA-IR**	r=0.-0928	r=0.1908	r=0.-0489	r=0.2321	r=0.2752	r=0.1708	r=0.0989	r=0.1303	r=0.1720
	p=0.369	p=0.063	p=0.636	p=0.023	p=0.007	p=0.096	p=0.338	p=0.206	p=0.094

Values show Pearson's r correlation coefficient together with correlation p-value. Correlations in bold type are significant with $p<0.00095$ (p value of 0.05 after Bonferroni correction for multiple comparisons); OGTT - oral glucose tolerance test (values for 0min and 120min); HbA1c –glycated hemoglobin; BMI – body mass index; FAT% (DXA) – percentage of body fat as measured by dual-energy X-ray absorptiometry; HOMA-IR – homeostatic model assessment of insulin resistance; a - $p<0.00095$.

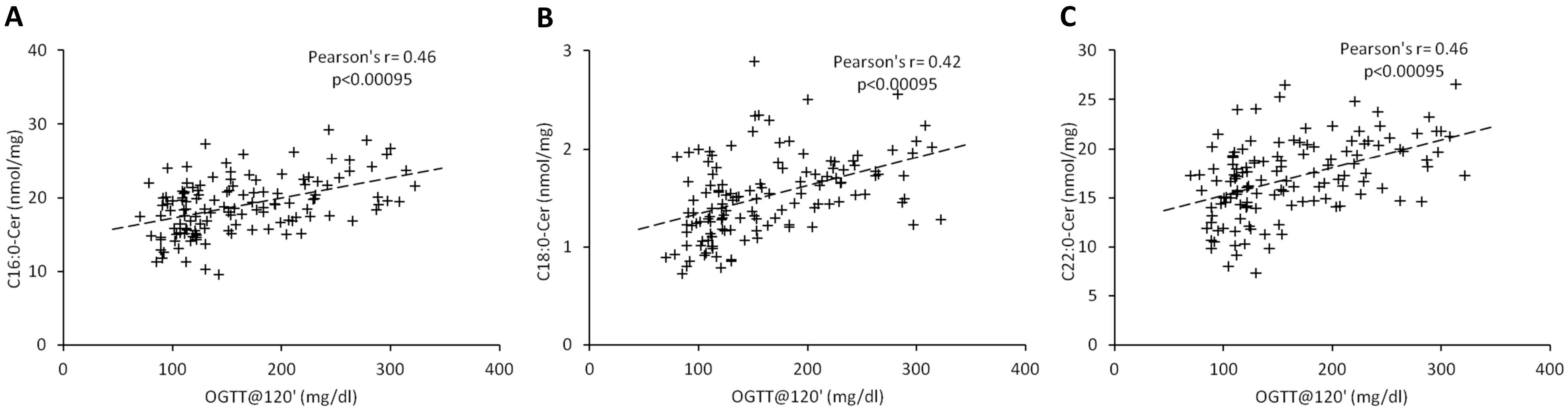

Figure S2. Correlation of C16:0 (Panel A), C18:0-Cer (Panel B) and C22:0-Cer (Panel C) with blood plasma glucose concentration at 120 min of OGTT test in combined group. Pearson's r correlation coefficient and correlation significance is given in graph inserts. OGTT – oral glucose tolerance test.
